# Dietary and other lifestyle characteristics of Cypriot school children: results from the nationwide CYKIDS study

**DOI:** 10.1186/1471-2458-9-147

**Published:** 2009-05-20

**Authors:** Chrystalleni Lazarou, Demosthenes B Panagiotakos, Christiana Kouta, Antonia-Leda Matalas

**Affiliations:** 1 Harokopio University, Department of Nutrition and Dietetics, Athens, Greece; 2 Cyprus University of Technology, School of Health Sciences, Nicosia, Cyprus

## Abstract

**Background:**

Dietary and lifestyle behaviors at young ages have been associated with the development of various chronic diseases. Schools are regarded as an excellent setting for lifestyle modification; there is a lack, however, of published dietary data in Cypriot school children. Thus, the objective of this work was to describe lifestyle characteristics of a representative segment of Cypriot school children and provide implications for school health education.

**Methods:**

The CYKIDS (Cyprus Kids Study) is a national, cross-sectional study conducted among 1140 school children (10.7 ± 0.98 years). Sampling was stratified and multistage in 24 primary schools of Cyprus. Dietary assessment was based on a 154-item semi-quantitative food-frequency questionnaire and three supplementary questionnaires, assessing dietary patterns and behaviors. Adherence to the Mediterranean diet was evaluated by the KIDMED index (Mediterranean Diet Quality Index for children and adolescents). Physical activity was assessed by a 32-item, semi-quantitative questionnaire.

**Results:**

Analysis revealed that 6.7% of the children were classified as high adherers, whereas 37% as low adherers to the Mediterranean diet. About 20% of boys and 25% of girls reported "not having breakfast on most days of the week", while more than 80% of the children reported having meals with the family at least 5 times/week. Some food-related behaviors, such as intake of breakfast, were associated with socio-demographic factors, mostly with gender and the geomorphological characteristics of the living milieu. With respect to physical activity, boys reported higher levels compared to girls, however, one fourth of children did not report any kind of physical activity.

**Conclusion:**

A large percentage of Cypriot school children have a diet of low quality and inadequate physical activity. Public health policy makers should urgently focus their attention to primary school children and design school health education programs that target the areas that need attention in order to reduce the future burden of metabolic disorders and chronic diseases.

## Background

During the past decades Cyprus has experienced marked socio-demographic changes [1] which have been followed by an accelerating pace of epidemiological transition and the emergence of risk factors for chronic diseases, such as obesity, in both children and adults [2,3], as well as deaths due to chronic diseases, such as cardiovascular disease (CVD) and cancer [4-6]. Epidemiological transition is generally attributed to major lifestyle changes, especially in dietary choices and physical activity, which lead to increased morbidity and mortality at population level, both in developed and less developed societies [7,8]. Well designed, effective health planning and interventions should be based on sound knowledge of the health status of a population through the evaluation of lifestyle behaviors (i.e. dietary choices, physical activity habits, smoking habits, etc), prevalence of major risk factors (i.e. obesity, hypertension, diabetes, dyslipidemias), and incidence rates of common chronic diseases. In Cyprus however, despite the improvements in health care services during the past years, published data on lifestyle characteristics for any group of the population, including children, are segmentary. Provided that, as it is widely acknowledged, the effectiveness of chronic diseases prevention might be maximized when it starts from early childhood [9,10], there is an emerging need for obtaining data on children's lifestyle habits. The CYprus KIDS (CYKIDS) study is a primary school population-based survey that aimed to assess lifestyle characteristics, i.e. dietary and physical activity patterns, in a representative sample of Cypriot children and provide needed information for public health preventive programs aimed at primary school children. The aim of the present work is to report results of the CYKIDS study on basic dietary and physical activity patterns in relation to selected sociodemographic factors and examine implications for public health programs.

## Methods

### Study population

The CYKIDS (CYprus KIDS) study is a nation-wide, cross-sectional survey that was conducted during the school year 2004–2005 and covered all the freely accessed districts of the Republic of Cyprus. Sampling was multistage and stratified by the number of students in each of the five provinces, as provided by the Ministry of Education (data available on request from the Department of Primary Education) and by place of residence (place of school was used as a proxy), urban or rural, as provided by the Cyprus Statistical Service [11].

A total of 1589 children of 4^th^, 5^th ^and 6^th ^grade (9–13 years, x = 11 ± 0.98) in 24 primary schools were identified for potential inclusion; 1140 agreed to participate (72% participation rate), representing 3.7% of the total population. All classes were of mixed ability. Diet quality was assessed in all children and the participating parents. We received 1068 responses from parents; only 72 less compared to children's responses.

A detailed description of the study design and dietary assessment methodology has been published elsewhere [12].

The research protocol and all means used were approved by the Ministry of Education and Culture (Department of Primary Education) as the law provides in Cyprus for the studies carried out in the school environment and during formal school hours. Informed consent was signed by the parent or the guardian of each participant.

### Dietary assessment

Dietary assessment was based on a semi-quantitative food-frequency questionnaire (FFQ), consisting of 154 food items (including all of the commonly used foods of the local Greek-Cypriot cuisine) and three supplementary questionnaires that evaluated other aspects of dietary habits. Specifically, a Food Groups Frequency Questionnaire (FGFQ), which evaluated on a four point scale the frequency of consumption of 15 food groups, a Short Eating Habits Behaviors & Beliefs Questionnaire (SEBBQ), which evaluated 8 psychological aspects of eating, using a four point Likert-type of scale, and a short Dietary Habits Questionnaire (SDHQ) that evaluated 19 core eating habits on a two-point of yes/no scale. Detailed description of these tools is provided elsewhere [12,13]. The questionnaires are available online at  or can be requested by the corresponding author.

The dietary questionnaires were administered to all students of each class during school hours, from February to June of 2005, by the same person- a qualified nutritionist, according to a written protocol describing a standardized methodology for dietary data collection.[12] Standard portion sizes that could be easily understood by children, such as the size of a fruit, were included in the questions, whereas for other foods, food models of the USA Dairy Council [14] and NASCO[15] were used as aids to visualize the regular portion. Food models were also used to help children recognize unfamiliar food terms.

Test-retest repeatability of the FFQ was tested by giving the same questionnaire in 100 children of the sample, within one month apart and was found to be good (Spearman rho correlation coefficient ranged from 0.180 to 0.670, with 95% items above 0.400 (indicating good reliability) and only 7 out of 154 items, i.e., 5%, were less than 0.200). Internal reliability was tested by Cronbach's alpha and found to be equal to 0.908, which indicates very good consistency of the FFQ. Validation against the short food groups' frequency questionnaire showed a mean coefficient of the association (Spearman's rho) between the two dietary tools of 0.237. Validation against a 24 hr recall is in future plans. The above figures indicate an adequate reliability and validation coefficients of the FFQ used. Food consumption frequencies among boys and girls are presented in the Results section.

### Assessment of Mediterranean diet patterns

The KIDMED index (Mediterranean Diet Quality Index for children and adolescents) was used to evaluate the overall quality of diet. This index has been developed by Serra – Majem et al [16] and includes 16 components that summarize the principles of the Mediterranean diet by an arithmetic score, which ranges from 0–12. According to authors, a score of 0–3 reflects a poor diet in relation to the Mediterranean diet principles, whereas values of 4–7 and values of 8–12, average and good adherence to the principles of the Mediterranean diet, respectively [16]. A more detailed description of the KIDMED index is provided elsewhere [12,16]

### Physical activity assessment

Children responded to a 32-item, semi-quantitative questionnaire, and assessed organized and free-time physical activity [17,18]; i.e. frequency and duration of everyday physical and sedentary activities on the weekdays, the weekend and on the previous day, using an 8-level answer scale ranging from "0" to "more than 8 hours" per day or week. Furthermore, for assessing the time spent to individual physical activities, such as bicycling, basketball, racket sports, volleyball, running, soccer, swimming e.t.c., a four-level answer scale ranging from "0 times per week" to "more than 6 times per week", was used. Questions about physical activity lifestyle and behaviors (e.g., commuting to school) were also included, having four qualitative frequencies as possible answers ranging from "rarely/never", to "always".

### Anthropometry and obesity definition

Children's height and weight were reported by parents via a short socio-demographic questionnaire sent out from the schools. Obesity and overweight among children were calculated using the International Obesity Task Force (IOTF) age and sex -specific Body Mass Index (BMI) cut – off criteria [19]. Parents' obesity and overweight percentages were also estimated from self reported values of body weight and height. BMI measures were used to define adult (parents) obesity (BMI ≥ 30 kg/m^2^) and adult overweight (BMI 25–29.9 kg/m^2^), according to the World Health Organization classification for adults. Further information is provided elsewhere [3].

### Socio-demographic variables

Socio-demographic characteristics such as age, gender, size of the family, (children)living in a refugee camp (yes/no), and parental relationship, were provided by the children who filled a respective questionnaire. Place of living was defined by using as a proxy the location of the school, as in Cyprus, is mandatory for children to attend the school of their area. Information regarding other demographic characteristics, that could not be answered with sufficient reliability by children, such as parents' age, educational level, whether parents ever living in a refugee camp, yearly income, and profession, were collected via a short questionnaire – attached to the consent form – that was completed by the parents. Family SES was defined by the InterCollege Research Center of Cyprus, based on parents' profession and educational level; the highest level of profession, reported by either parent, was used as a proxy of the family's SES level, along with family income. A similar procedure for defining parental educational level has been used by Velde et al. [20].

### Statistical analysis

Continuous variables are presented as mean ± SD, whereas qualitative variables are presented as absolute and relative frequencies. Normality of variables' distribution was tested by Kolmogorov- Smirnov test. Associations between gender and normally distributed variables were tested by Student's independent t- test, or by the Mann-Whitney U test for the non-normally distributed variables. Associations between categorical variables were tested using contingency tables and the calculation of the chi-square test without Yate's correction.

Missing cases were within the accepted limits of 10–15%. No significant patterns with regards to any socio-demographic factors were evident in missing cases.

All reported p-values are based on two-sided tests and compared to a significance level of 5%. SPSS 13.0 software (Statistical Package for Social Sciences, Chicago, IL, USA) was used for all statistical calculations.

## Results

### Demographic characteristics of the participants

Mean age was 10.7 years for both boys and girls (p = 0.827) (Table 1). Distribution of socio-demographic characteristics including school grade, place of residence, geographic characteristics of place of residence, SES, ethnicity, refugee status, type of house, and number of household inhabitants was similar in boys and girls.

**Table 1:** Descriptive characteristics of the sample

	Boys	Girls	P
**Age**	10.68 (0.96)	10.67 (0.99)	0.827
**Grade**			0.705
*4*^*th*^	183 (34.5)	195 (32.1)	
*5*^*th*^	174 (32.8)	206 (33.9)	
*6*^*th*^	174 (32.8)	206 (33.9)	
**Place of living**			0.602
*Urban*	291 (54.8)	342 (56.3)	
*Rural*	240 (45.2)	265 (43.7)	
**Geomorphologic characteristics of residence**			0.590
*Plain*	415 (78.2)	466 (76.8)	
*Sub-mountainous*	36 (6.8)	51 (8.4)	
*Mountainous*	80 (15.1)	90 (14.8)	
**Socioeconomic level**			0.772
*High*	87 (21.2)	111 (21.9)	
*Average*	171 (41.7)	219 (43.3)	
*Low*	152 (37.1)	34.8 (176)	
**Ethnicity**			0.667
*Greek*	377 (88.7)	461 (88.5)	
*Foreigners*	14 (3.3)	13 (2.5)	
*Mixed*	34 (8.0)	47 (9.0)	
**Type of residence**			0.263
*Apartment*	65(12.9)	84(14.3)	
*House with a yard*	403(80.3)	476(81.1)	
*House without a yard*	34(6.8)	27(4.6)	
**Residence in a refugee camp**	31(6.4)	43(7.6)	0.472
**Number of inhabitants in home**			0.518
≤ *3–4*	191(42.2)	227(41.1)	
*5–6*	218 (48.1)	281(50.9)	
≥ *7*	44(9.7)	44(4.4)	
**Body Mass Index Classification- Children**			0.081
*Normal weight*	277 (75.1)	358 (78.9)	
*Overweight*	70 (19.0)	83 (18.3)	
*Obese*	22 (6.0)	13 (2.9)	
**Parental obesity status**			0.085
*Both lean*	131 (33.3)	137 (29.2)	
*Both obese*	90 (22.9)	87 (18.6)	
*Father obese/mother lean*	146(37.2)	203(43.3)	
*Father lean/mother obese*	26(6.6)	42(9.0)	

Data are presented as mean (SD) and categorical variables as frequencies and percentages in parentheses.

### Lifestyle characteristics of the participants

Table 2 presents results from analyses carried out to examine differences between boys and girls with regards to the frequency of consumption of several food groups and individual food items. Significant differences were observed for 14 food groups or individual items; boys reported more frequent consumption of eggs, meat, poultry, delicatessen, fish, legumes, nuts, puff pastry, fresh legumes, potatoes, olives, candy and soda, whereas 'low glycemic foods' were consumed more frequently by girls. Evaluation of the overall quality of diet (using the KIDMED index) showed no difference between genders (P = 0.097) (Table 2). Further details are provided elsewhere [12].

**Table 2:** Food consumption and diet quality by frequency category among boys and girls

Food groups	Frequency categories	Boys	Girls	p*
**N**		533	607	
**DAIRY**				
*Milk*	≥ 7 times/week	88.0	89.1	0.602
*Semi Skimmed milk*	≥ 7 times/week	66.0	69.5	0.217
*Yogurt*	≥ 7 times/week	45.1	42.2	0.346
**MEAT & ALTERNATIVES**				
*Eggs*	≥ 2 times/week	52.8	44.8	0.010
*Meat (excluding poultry, mixed dishes)*	≥ 2 times/week	29.0	18.6	<0.001
*Poultry & rabbit meat*	≥ 2 times/week	25.3	15.1	<0.001
*Delicatessen*	≥ 5 times/week	28.2	16.2	<0.001
	2–4 times/week	18.6	15.1	
	≤ 4 times/month	53.2	68.7	
*Fish & seafood*	≥ 2 times/week	30.0	20.6	<0.001
*Legumes*	≥ 5 times/week	18.1	11.1	0.005
	2–4 times/week	12.7	14.1	
	≤ 4 times/month	69.2	74.8	
*Nuts*	≥ 2 times/week	29.3	18.2	<0.001
**CEREALS, GRAINS &PRODUCTS**				
*White bread*	≥ 7 times/week	70.0	69.9	0.960
*Whole grain bread*	≥ 7 times/week	21.2	19.5	0.502
*Breakfast cereals*	≥ 5 times/week	51.2	48.2	0.324
*Puff pastry*	≥ 5 times/week	21.3	13.4	<0.001
	2–4 times/week	12.7	9.4	
	≤ 4 times/month	66.0	77.3	
*Pasta*	≥ 5 times/week	14.0	10.6	0.100
*Bulgur*	≥ 5 times/week	35.1	31.8	0.260
*Rice dishes*	≥ 5 times/week	32.6	31.5	0.695
**FRUITS**				
*Fresh fruit juices*	≥ 7 times/week	35.8	36.3	0.867
*Fruits*	≥ 2/day	73.8	74.2	0.860
**VEGETABLES**				
*Vegetables*	≥ 2/day	46.1	41.8	0.159
*Fresh legumes, other seasonal consumed vegetables*	≥ 5 times/week	12.1	7.4	0.010
*Potatoes & potatoes dishes*	≥ 2 times/week	41.7	35.0	0.028
*Olives*	≥ 5 times/week	31.1	22.4	0.001
**OTHER GROUPINGS**				
*Potato chips*	≥ 7 times/week	25.1	22.0	0.222
*Biscuits*	≥ 7 times/week	48.6	51.4	0.521
*Candy*	≥ 7 times/week	21.8	15.1	0.005
*Soda*	≥ 7 times/week	31.3	21.9	<0.001
*Chocolates*	≥ 7 times/week	25.1	21.1	0.121
	≥ 5 times/week	45.6	43.3	0.442
*Pizza*	≥ 2 times/week	45.7	41.1	0.123
Low glycemic index foods**	≥ 3 times/week	59.2	67.4	0.031
*Medium glycemic index foods*	≥ 3 times/week	66.1	65.3	0.854
*High glycemic index foods*	≥ 3 times/week	60.9	58.4	0.472
**Quality of diet (as assessed by KIDMED score)**				0.097
*Poor quality diet: score 0–3*		44.8	55.2	
*Average quality diet: score 4–7*		40.9	59.1	
*Good quality diet: score 8–12*		55.4	44.6	

Data are presented as percentages.

* From chi-square.

Data on basic food-related behaviors, such as types and location of meals, having meals with family company and methods of cooking are presented in Table 3. About 20% of boys and 25% of girls reported "not having breakfast on most days of the week", while more than 80% of the children reported having at least 5 times/week meals with the family.

**Table 3:** Meal patterns among boys and girls

	Boys	Girls	P*
***N***	533	607	
Intake of breakfast ≥ *5 times/week vs. ≤ 4 times/week*(%)	81.3	75.3	0.019
	18.7	24.7	
Number of main meals & snacks daily ≤ *3/day vs. ≥ 4/day*(%)	39.1	35.9	0.301
	60.9	64.1	
Number of snacks per day ≤ *1 per day vs. ≥ 2 per day*(%)	26.5	22.3	0.116
	73.5	77.7	
Eating outside home or order fast food in past 2 days *0 times vs. ≥ 1 times*(%)	54.2	60.8	0.033
	45.8	39.2	
Have meals with family ≥ *5 times/week vs. ≤ 4 times/week*(%)	84.6	82.1	0.261
	15.4	17.9	
Have meals alone ≥ *5 times/week vs. ≤ 4 times/week*(%)	41.8	33.0	0.030
	58.2	67.0	
Have lunch at school *4 times/week vs. ≤ 3 times/week*(%)	14.9	10.5	0.032
	85.1	89.5	
Eat fried food ≥ *3 times/week vs. ≤ 2 times/week*(%)	49.4	44.8	0.112
	50.6	55.2	
Eat grilled food ≥ *3 times/week vs. ≤ 2 times/week*(%)	41.4	36.7	0.116
	58.6	63.3	

From chi-square, df = 1

The relationship of socio-demographic chracteristics to these food-related behaviors was also examined. Besides gender (Table 3), significant relations were found for the geomorphological traits of the living milieu (plain, sub-mountainous, mountainous): compared to children living in plains, children in mountainous and sub -mountainous regions ate less frequently outside home (i.e. in fast food outlets or restaurants (p < 0.001) and with family (p = 0.001), but ate fried food at least 3 times a week (p = 0.002). Additionally, children from mountainous areas ate breakfast over 5 times/week (p = 0.005) and ate more frequently during the course of the day (≥ 4 times/day).

With respect to the other socio-economic factors, differences were found by refugee status, socio-economic status, ethnicity, and family size. Specifically, children living in refugee camps, ate with their family less frequently (p < 0.001) while they also ate fried food less frequently (p = 0.012); children belonging to low SES had lunch at school more frequently (p = 0.004), and ate fried food more frequently (p = 0.035); children belonging to minorities were more likely to eat alone (p = 0.007), while those of families with four or less members,, ate more frequently outside home (p = 0.054).

With respect to dietary quality, no significant relationships were found for any of the above socio-economic factors.

### Physical activity levels and sedentary patterns levels

Associations between physical activity habits and the socio-demographic factors listed in Table 1 revealed that the most important and consistent differences were those observed between the two genders. Physical activity and sedentary behaviour are therefore presented by gender, as well (Table 4).

**Table 4:** Comparison of physical activity pursuits by gender

Percentage	Boys	Girls	p
Have private lessons > 2 hours/day	51.8	55.1	0.280
Do homework ≥ 2 hours/day	28.6	38.5	<0.001
Watch TV- DVD ≥ 2 hours/day	65.8	61.4	0.280
Watch TV- DVD ≥ 2 hours in weekend days	77.1	79.2	0.700
Play electronic games or use computers ≥ 1 hours/day	69.8	41.5	<0.001
Play electronic games or use computers ≥ 1 hours in weekend days	74.2	50.3	<0.001
Did not participate at all in sports the previous day	34.6	65.4	<0.001
Did not participate at all in any kind of physical activity the previous day	22.6	24.8	0.385
Do any kind of home chores at least 1/2 hour/day	73.3	93.2	<0.001
Do in any kind of out of home chores at least 1 hour/week	37.3	32.3	0.086
Mean (SD) Hours			
Have private lessons at weekdays	2.56 (1.93)	2.62 (1.83)	0.151
Do homework at weekdays	1.70 (1.19)	1.95 (1.32)	0.079
Watched TV-video the day before	2.42 (2.06)	2.32 (1.96)	0.317
Watched TV-video at weekends	4.25 (2.63)	4.18 (2.64)	0.806
Played with electronic games, computer the day before	1.15 (1.73)	0.64 (1.16)	<0.0001
Played with electronic games, computer at weekends	2.79 (2.68)	1.59 (2.05)	<0.001
Engaged in sports after school the day before	1.32 (1.58)	0.66 (1.06)	<0.0001
Engaged in physical activity after school the day before	1.50 (1.74)	1.17 (1.35)	0.001
Engaged in home chores/week	2.79 (6.52)	5.69 (8.37)	<0.0001
Engaged in outside home chores/week	2.31 (6.05)	1.90 (4.31)	0.219

Data are expressed as mean (SD) or percentages.

In specific, Table 4 shows the percentage of children who were engaged in certain physical or sedentary activities, as well as the mean duration of the corresponding behaviours. Boys generally seem to have higher level of physical activities and spend more time in electronic games, while girls spend more time in homework and do more home chores. However, about one fourth of the children reported that they did not have any kind of physical activity and one third of boys and two thirds of girls that do not participate in any kind of sports in a typical day. Moreover, there are no gender differences regarding the time spent in TV viewing in week days (p = 0.317) or weekends (p = 0.806), as well as the percentage of children who spent more than 2 hours per day in weekdays (p = 0.280) or in weekend days (p = 0.700) watching TV (Table 4).

## Discussion

In this paper we present the aims, methodology and basic findings of the CYKIDS study, a nationwide, epidemiological study that examines the dietary habits and physical activity patterns of school-aged children in Cyprus. Our aim was to evaluate school children's dietary habits and physical activity patterns in relation to selected socio-demographic factors and examine implications for public health programs.

Our survey revealed several negative trends with regards to children's diet. A large percentage of participants reported frequency of consumption of fruit, vegetables and whole grain products that are well below recommended levels. Adequate intake of these foods is important in achieving optimal dietary quality as well as in preventing obesity and other risk factors for chronic disease [21].

Certain dietary practices, such as eating breakfast and having meals with family have been associated with sound diet and prevention of childhood obesity [22,23]. It is therefore of concern, that a fifth of boys and a quarter of girls do not eat breakfast most days. Similar figures for skipping breakfast (i.e. 10%–30%) have been reported by previous studies conducted in the United States and Europe [24]. It is encouraging however, that the vast majority of children eat most of their meals with family members. Several observational studies have shown that parental presence at meal time is associated with better dietary habits as well as, decreased frequency of skipping breakfast [25-29]. Family-based interventions are therefore being regarded as effective means for the prevention of childhood obesity and the promotion of healthy lifestyles [30,31]. Family cohesiveness is still very strong in Cyprus [32] and it is still common that grandparents take the care of children when they finish classes. Grandparents often prepare lunch and/or dinner on weekdays, and even at weekends, for their children and grandchildren. Thus, children eat home-made meals with their family. A mountainous, rural environment was found to be a significant factor in determining children's eating behaviour. In general terms, living in mountainous or sub-mountainous regions was found to be associated with healthier behaviours, such as having breakfast regularly, joining family meals, and consuming fast food less often. Mountains are generally acknowledged in Europe as a living milieu where traditional ways of living have not been abandoned and family cohesiveness has been preserved. Family cohesion has been shown to be predictive of positive dietary behaviours [33]. Gender also appeared to influence food-related behaviours, such as having breakfast. Similarly to previous studies [34], we found that girls are more likely to skip the first main meal of the day, in all likelihood as a means to lose weight [35]

It is generally acknowledged that diet should be assessed holistically taking into account foods and food groups consumed as well as, dietary practices, such as meal patterns[36]. We used the KIDMED index to assess the overall quality of children's diet in relation to the Mediterranean diet model. Our findings point to no significant differences between boys and girls. But noticeably, only 6.7% of the children were classified as high adherers to the Mediterranean diet, whereas 37% had a poor KIDMED score. A previous study conducted in Crete [37] that evaluated adherence to the Mediterranean diet among 690 children and adolescents (7–18 years) by using a modified KIDMED index, reported results similar to ours for the lowest KIDMED category, i.e.27.9% of the children had a poor Mediterranean diet score, but the percentage who scored highest was 4 times higher, i.e. 28.5% as opposed to 6.5% in our study. A survey conducted by Kontogianni et al [38] in a representative sample of 1305 Greek children and adolescents that assessed adherence to Mediterranean diet by applying the same tool, the KIDMED index also found, that only a small percentage of children (11.3%) and adolescents (8.3%) obtained an optimal KIDMED score (i.e. ≥ 8 points).

It seems that, there has been a shift from traditional healthy diets towards unhealthy ones. This phenomenon should be further explored by future studies to reveal the underlying causes.

Regarding differences in levels of physical activity and sedentary patterns between boys and girls, the results of our study were similar to those of other studies conducted in Cyprus [39] and abroad [40], which show that boys have higher levels of physical activity, compared to girls. According to our study, a large percentage of both boys and girls have less than the recommended levels of physical activity (i.e. even though the average amount of physical activity is more than 60 minutes daily in both genders, one fifth of boys and one quarter of girls are completely sedentary, after school. Even though factors associated with levels of physical activity in Cypriot children are have been investigated [41,42], differences between genders however, have not been evaluated yet and this warrants further investigation.

Average TV viewing time was high compared to the American Academy of Pediatrics recommended daily time[43], especially at weekends, and moreover, the vast majority of children failed to meet these recommendations. Our data are comparable with the findings of a previous study conducted among 1337 Cypriot children (11–12 years) during the period 1998–1999, which showed that 67.4% of boys and 68.3% of girls spent more than 2 hours/day watching TV [44]. These results call for specific measures to target TV viewing and further investigations regarding correlates and clustering of this particular behavior with other lifestyle behaviors.

### Limitations and strengths

The present study has certain limitations which should be taken into account before drawing generalisations from its results. First, it was a cross-sectional study. Therefore, no conclusions can be made about plausible causes, but rather, indications can be extracted, valuable in future investigations. Dietary and physical activity data were based on self-reports. Although we made every effort to get as accurate data as possible, there is a possibility that misreporting has occurred, which might have influenced our findings. Literature reports that food underreporting is usually associated with gender and weight status [45,46]. Girls and overweight children are more prone to underreporting, underreporting high energy dense, and low nutrient dense, foods, in particular[45,46]. Assessment of obesity status was based on parental reported heights and weights, but this practice has been previously used with satisfactory results [47,48].

Some of the strengths of the present work deserve a comment. First, its nation-wide character allows to draw generalizations from the findings for the Cypriot population. It also evaluates a wide array of socio-demographic parameters for their association to children's diet and physical activity. Finally, it provides implications and suggestions valuable in public health policies and remedial actions.

### Implications

The data presented here provide information which can be useful in public health programs. Schools are regarded as excellent settings for providing health education and lifestyle intervention programs to children [49]. The Ministry of Education, educators, public health professionals and parents should be aware of these findings and explore new ways to encourage regular meal consumption and urge more children to eat breakfast regularly, have meals with their family and have fewer meals away from home. In particular, health care professionals and dieticians have an important role to play in encouraging the adoption of regular family meals by highlighting the benefits of this habit, suggesting strategies and helping families in setting progressive and achievable goals. Furthermore, efforts should be made to increase consumption of vegetables, fruit, legumes, and whole grain products and limit consumption of sodas and high glycemic foods.

The benefits of the Mediterranean Diet should be highlighted and concerted public health actions should be undertaken in order to promote this traditional prototype of healthy eating.

In the light of the above finding, that some important food behaviours are related to certain socio-demographic factors and especially to gender and the geomorphological characteristics of living environment, public health programs should be differentiated accordingly and provide for sustaining the positive dietary behaviours in the groups that have preserved traditional foodways, while giving special emphasis and planning targeted actions in those populations, that are more susceptible in adopting "unhealthy" dietary behaviours.

New research should be undertaken to reveal which are the factors that influence the adoption of regular meal patterns and optimal food consumption patterns. As for physical activity, our results suggest that future intervention programs should specifically target girls who were found to be the less active.

## Conclusion

This is the first study which reports on dietary habits of Cypriot school children and showed that at least one third of Cypriot children have adopted poor dietary habits, whereas about one fourth, do not have any kind of physical activity on a typical day. It is noteworthy that more than 60% of participants watch TV- DVD at least 2 hours/day. Boys reported higher level of physical activities and spend more time in electronic games, while girls spend more time in homework and do more home chores. The gender-specific differences in lifestyle factors that were observed, point to the need for specific public health programs for this population group. Public health policy makers and other health care professionals should urgently focus their attention to school children in order to reduce the burden of metabolic disorders and chronic diseases in the future.

## Competing interests

The authors declare that they have no competing interests.

## Authors' contributions

CL designed the study, obtained the funding, performed the data analysis, interpreted the results and wrote the paper. DBP supervised the design of the study, the statistical analyses, contributed to the presentation and interpretation of the results and critically reviewed the paper. CK critically reviewed the paper and A-LM contributed to the presentation and interpretation of the results and critically reviewed the paper. All authors contributed to the final version of the manuscript.

## Pre-publication history

The pre-publication history for this paper can be accessed here:


